# The association between midlife serum high-density lipoprotein and mild cognitive impairment and dementia after 19 years of follow-up

**DOI:** 10.1038/s41398-018-0336-y

**Published:** 2019-01-18

**Authors:** Thomas Svensson, Norie Sawada, Masaru Mimura, Shoko Nozaki, Ryo Shikimoto, Shoichiro Tsugane

**Affiliations:** 1 Epidemiology and Prevention Group, Center for Public Health Sciences, National Cancer Center, 5-1-1 Tsukiji, Chuo-ku, Tokyo, 104-0045 Japan; 2 Department of Neuropsychiatry, Keio University School of Medicine, 35 Shinanomachi, Shinjuku-ku, Tokyo, 160-8582 Japan; 3 Clinical Biotechnology, Center for Disease Biology and Integrative Medicine, Graduate School of Medicine, The University of Tokyo, 7-3-1 Hongo, Bunkyo-ku, Tokyo, 113-8656 Japan; 4 Department of Clinical Sciences, Lund University, Skåne University Hospital, Malmö, Sweden

## Abstract

A third of dementia cases could be attributable to modifiable risk-factors. Midlife high-density lipoprotein cholesterol (HDL-C) is a measure which could help identify individuals at reduced risk of developing age-related cognitive decline. The Japan Public Health Centre-based prospective (JPHC) Study is a large population-based cohort which started in 1990. This study included 1299 participants from Saku area in Nagano prefecture. Participants had HDL-C measured in 1995–1996, and underwent a mental health screening in 2014–2015. Of these, 1114 participants were included in MCI analyses, and 781 participants were included in dementia analyses. Logistic regression models were used to determine odds ratios (OR) and 95% confidence intervals (CI) for the association between HDL-C quartiles and MCI and dementia, respectively. For dementia analysis, quartiles 2–4 were collapsed due to low number of cases. Missing data was addressed through multiple imputations. There were 386 cases of MCI and 53 cases of dementia. Compared to the lowest HDL-C quartile, the highest HDL-C quartile was significantly inversely associated with MCI (OR = 0.47, 95% CI, 0.28–0.79) in the multivariable analysis. High HDL-C (quartiles 2–4) was inversely associated with dementia compared to low HDL-C (quartile 1) (OR = 0.37, 95% CI, 0.16–0.88). This study has found that high midlife HDL-C levels are inversely associated with both late-life MCI and dementia in a Japanese population.

## Background

It is estimated that one third of dementia disease cases could be attributable to modifiable risk factors.^1^ A key to intervention and prevention is thus the early identification of measures associated with both dementia and mild cognitive impairment (MCI), a “state intermediate between normal cognition and dementia”.^2^ One such measure is serum high-density lipoprotein cholesterol (HDL-C), a potentially stronger predictor of age-related cognitive decline^3^ and Alzheimer’s disease (AD) in the elderly^4^ than other lipid measures. High levels of HDL-C are inversely associated with both MCI^5^ and dementia,^4,6^ albeit with some discrepant findings reporting no association between HDL-C and either cognitive function,^7–10^ MCI,^11,12^ or dementia.^13–18^ Such ambiguity of results could be due to the timing of the HDL-C measure in relation to the cognitive impairment. Studies investigating the association between HDL-C and cognitive function have measured HDL-C at old age and often concomitantly with the assessment of cognitive function. Cholesterol levels decline prior to development of dementia,^19^ and studies reporting HDL-C levels measured in late-life or at the time of disease may thus possibly suffer from reverse causation bias. Measuring HDL-C at midlife, i.e., years before disease onset may therefore allow for early intervention and lifestyle modification.

The primary aim of the present study was thus to investigate, in a prospective Japanese general population cohort, the association between midlife HDL-C and late-life MCI and dementia, respectively. We hypothesize that high levels of midlife HDL-C will be inversely associated with both late-life MCI and dementia.

## Methods

### Study population

The Japan Public Health Center-based prospective (JPHC) Study was started in 1990 and conducted in two cohorts, one initiated in 1990 (cohort I) and the other in 1993 (cohort II). The study design has been described in detail elsewhere.^20^ JPHC Study participants were identified by the population registries maintained by the local municipalities in 11 public health center (PHC) areas. At baseline, there were 140,420 individuals identified in the study population (eFig. 1). The participants of the present study were 12,219 individuals who in 1990 were aged 40–59 years and registered as residents of Saku PHC area (Nagano prefecture). Following the exclusion of those who had moved out of the Saku PHC area, had died, or did not respond to the JPHC Study baseline questionnaire (*n* = 3392), there were 8827 eligible participants who in 2014–2015 were invited to participate in a mental health screening. A total of 1299 individuals attended the screening. Of these, 1167 individuals were eligible for analyses in the present study following the exclusion of those with cardiovascular disease (CVD) (*n* = 19) defined through self-report or confirmed through hospital registers at the time of measuring HDL-C. Additionally, we excluded those who were diagnosed with depression at the time of the mental health screening (*n* = 113).

For the purpose of MCI analyses, we further excluded 53 individuals who were diagnosed with dementia at the time of screening, leaving 1114 participants of which 651 participants had complete information on all variables. For the purpose of dementia analyses, 386 persons with confirmed MCI were excluded, thereby leaving 781 participants for analyses of which 455 participants had complete information on all variables.

Surveys of JPHC study participants were conducted on three occasions and included detailed questionnaires with information on socioeconomic and lifestyle factors. The present study used information from the baseline (1990) and 5-year follow-up survey (1995).

All participants provided written informed consent to take part in the mental health screening in 2014–2015, and the study has been approved by the Institutional Review Board of the National Cancer Center (approval number: 2013–096).

### Serum lipids

The details on obtaining participants’ lipids have been previously described.^21^ In brief, information was available for non-fasting total cholesterol (TC) and HDL-C, with TC measured using an enzymatic method. Fasting status at time of HDL-C measurement does not impact HDL-C results.^22^

Data on HDL-C was collected around the time of the 5-year follow-up survey for 703 and 493 of the 1167 participants eligible for MCI and dementia analyses, respectively. The mean age of study participants when collecting HDL-C data was 54.4 years (SD: 5.5 years) for MCI analyses, and 54.1 years (SD: 5.6 years) for dementia analyses. Triglycerides (TG) were available for 699 (MCI) and 490 (dementia) participants. Low-density lipoprotein cholesterol (LDL-C) was calculated for 689 (MCI) and 482 (dementia) participants using the Friedewald formula [TC-HDL-(TG/5)], excluding individuals with triglycerides (TG) > 4.52 mmol/l.

The HDL-C levels of complete cases were categorised into analyses-specific quartiles.

### Mild cognitive impairment and dementia

Cognitive function was assessed by experienced neuropsychologists using the Mini-Mental State Examination (MMSE),^23^ the Wechsler Memory Scale Revised (WMS-R) logical memory I/II subtest,^24^ the clock drawing test,^25^ and the Clinical Dementia Rating (CDR) Scale.^26^ Depressive symptoms were assessed using the Center for Epidemiologic Studies Depression Scale (CES-D),^27^ and the Patient Health Questionnaire-9 (PHQ-9).^28^ Participants’ cognitive function was subsequently categorised in accordance with criteria used in the Japanese Alzheimer’s Disease Neuroimaging Initiative (J-ADNI) project^29,30^where MCI was defined as amnestic MCI as originally presented by Petersen et al.^31^ Memory impairment was assessed based on an education-adjusted score below the cut-off level on the WMS-R Logical Memory II test (education for 0–9 years was 2, for 10–15 years was 4, and for >15 years was 8). The MMSE cut-off point for dementia was set at 23. A trained psychiatrist combined the neuropsychological assessment with a clinical interview to determine the final diagnosis in accordance with the Diagnostic and Statistical Manual of Mental Disorders, 4th. Edition Text Revision (DSM-IV-TR) criteria.^32^

### Statistical analyses

Differences in baseline characteristics between HDL-C quartiles, as well as between participants with complete and missing data, were determined using the Chi-square test for categorical variables, analysis of variance (ANOVA) for age, BMI and LDL-C, and Welch’s ANOVA for TG. The Pearson correlation coefficient was used to determine correlation between potential auxiliary variables in multiple imputation and missing status for the main exposure (HDL-C).

Logistic regression models were used to determine odds ratios (OR) and 95% confidence intervals (CI) for the association between HDL-C and MCI and dementia, respectively. Model 1 was adjusted for age (continuous) at the time of mental health screening, sex, and education (junior high school, high school, or college/vocational school/other). Model 2 was additionally adjusted for alcohol consumption (<150 or ≥150 g ethanol per week), smoking status (non-smoker, past smoker, or current smoker), and body mass index (BMI) (continuous in kg/m^2^) from measures obtained at the health check-up. Model 3 was further adjusted for hypertension (systolic blood pressure ≥140 mmHg or diastolic blood pressure ≥90 mmHg using health check-up data), self-reported history of diabetes mellitus (yes/no), use of cholesterol lowering medication (yes/no), LDL-C (continuous), and TG (continuous).

Statistical analyses were performed using Stata (Stata version 14.0 SE, StataCorp LP, College Station, TX, USA). *P*-values were two-tailed and considered significant if *p* < 0.05.

#### Post-hoc analyses

For dementia analysis, HDL-C quartiles 2–4 contained only a small number of cases, and the results for the association between HDL-C and dementia revealed comparable effect sizes for HDL-C quartiles 2–4 compared to the referent quartile 1. Consequently, quartiles 2–4 were collapsed thereby allowing for a dichotomous HDL-C variable used only in dementia analysis.

### Missing data

Data was missing for 41.6% and 41.7% of the participants in MCI and dementia analyses, respectively. eTable 1 shows differences in baseline characteristics between those with complete data and those with missing data for at least one of the variables in the final regression model. Differences between those with complete vs. missing data indicate that data was not missing completely at random. Under the assumption that data was missing at random, each missing value was imputed 100 times (*m* = 100) by multiple imputations using chained equations. Data was imputed separately for each outcome (MCI and dementia), and the imputation models included all covariates used in the final multivariable logistic regression model, as well as the respective outcomes. The continuous variables BMI, HDL-C, LDL-C, and TG were all transformed prior to imputation (log-transformation for BMI and HDL-C, square root transformation for LDL-C and TG), and back-transformed after imputation. One participant had an improbable TG level of “0” which was replaced as “missing” for subsequent imputation. No interactions were considered in the imputation models.

Failure to attend health check-up or health screening could be considered predictive of missing cholesterol levels. As such, variables representing attendance/non-attendance to health check-up examinations or screenings (i.e., monitoring blood pressure, blood tests, ECG, fundoscopy, chest X-ray, sputum cytology, photofluorography, upper gastro-intestinal endoscopy, faecal occult blood test, barium enema, colonoscopy, and for women also mammogram or the Papanicolaou smear) were checked for consideration as possible auxiliary variables. Each of these variables correlated weakly (<0.33) with missing HDL-C status, and were therefore not included as auxiliary variables in the imputation models.

### Role of the funding source

The sponsors had no role in study design; in the collection, analysis, and interpretation of data; in the writing of the report; or in the decision to submit the paper for publication.

### Availability of data, materials, and code

We cannot publicly provide individual data due to participant privacy, according to ethical guidelines in Japan. Additionally, the informed consent we obtained does not include a provision for publicly sharing data. Qualifying researchers may apply to access a minimal dataset by contacting Dr. Shoichiro Tsugane, Principal Investigator, Epidemiology and Prevention group, Center for Public Health Sciences, National Cancer Center, Tokyo, Japan, at stsugane@ncc.go.jp. Alternatively, please contact the Office of the JPHC Study Group at jphcadmin@ml.res.ncc.go.jp or stsugane@ncc.go.jp.

## Results

### Mild cognitive impairment

Compared to individuals included in complete case analysis, those with missing data for at least one of the variables included in the final models for MCI analyses were younger, more likely to be men, have higher education, more likely to be smokers, have higher mean TG levels and lower mean HDL-C levels (eTable 1). The variables with the highest prevalence of missing data (36.9%–38.3%) were related to the health check-up.

The mean HDL-C level for participants in MCI analyses was 1.54 mmol/l (SD: 0.40 mmol/l). The mean age at the time of measuring cholesterol was 54.4 years (SD: 5.5 years) and the mean age at the time of the mental health screening was 73.5 years (SD: 5.6 years). At the mental health screening, 386 participants were diagnosed with MCI, of which 225 were complete cases.

Baseline characteristics for complete cases according to HDL-C quartile show that those in the highest quartile were less likely to be men, less likely to be smokers, had lower mean BMI, and lower mean TG and LDL-C levels (Table 1).

**Table 1:** Baseline characteristics according to midlife serum high-density lipoprotein cholesterol (HDL-C) quartiles for MCI analyses

Characteristics	Serum HDL-C quartile (mmol/l)				*p* value^a^	Missing data (%)
	Quartile 1 (<1.29)	Quartile 2 (1.29–1.50)	Quartile 3 (1.53–1.76)	Quartile 4 (≥1.78)		
Proportion of all participants (%)	15.7	14.0	15.2	13.6		41.6
Age at screening [mean (years ± SD)]	73.5 ± 5.5	73.9 ± 6.1	73.2 ± 5.4	73.4 ± 5.3	n.s.	0
Men (%)	55.4	45.5	27.2	23.2	<0.001	0
Education (%)					n.s.	3.6
Junior high school	32.6	33.3	33.7	25.8		
High school	54.3	50.6	49.1	52.3		
College/vocational school, University or Other	13.1	16.0	17.2	21.9		
Alcohol consumption (%)					n.s.	0
<150 g ethanol per week	77.7	73.1	84.0	80.8		
≥150 g ethanol per week	22.3	26.9	16.0	19.2		
Smoking status (%)					<0.001	4.1
Non-smoker	63.4	69.9	82.8	85.4		
Past smoker	10.9	15.4	7.1	7.3		
Current smoker	25.7	14.7	10.1	7.3		
Body mass index (kg/m^2^ ± SD)	24.5 ± 2.8	23.6 ± 2.4	23.4 ± 2.5	22.5 ± 2.4	<0.001	36.9
History of diabetes (%)	4.0	3.2	1.2	0.7	n.s.	0
Hypertension^b^ (%)	28.6	27.6	27.2	21.2	n.s.	36.9
Using cholesterol lowering medication (%)	1.1	3.2	3.0	5.3	n.s.	0
Triglycerides [mean (mmol/l ± SD)]	1.8 ± 0.8	1.3 ± 0.6	1.2 ± 0.6	1.0 ± 0.5	<0.001	37.4
LDL-C [mean (mmol/l ± SD)]	3.2 ± 0.8	3.3 ± 0.8	3.4 ± 0.8	3.1 ± 0.8	0.0131	38.3

*LDL-C* low-density lipoprotein cholesterol

^a^Analysis of Variance (ANOVA) for age at screening, body mass index, and LDL-C; Welch’s ANOVA for triglycerides; Chi-square test for categorical variables

^b^Hypertension is defined as systolic ≥140 mm Hg or diastolic ≥90 mm Hg

Results of complete-case analyses are reported in eTable 2. Following imputation of missing values, the highest HDL-C quartile was significantly inversely associated with MCI compared to the lowest HDL-C quartile (OR = 0.47, 95% CI, 0.28–0.79) (Table 2). This association and the significant trend of decreasing ORs with each increasing HDL-C quartile persisted with only minimal attenuation in all three statistical models.

**Table 2:** The association between midlife serum high-density lipoprotein cholesterol (HDL-C) levels and mild cognitive impairment (MCI) by multiple imputations using chained equations (*n* = 1114, *m* = 100)

	HDL-C levels (mmol/l)^a^				
	<1.29	1.29–1.50	1.53–1.76	≥1.78	*P* for trend
MCI					
No. (Events)^b^	175 (69)	156 (62)	169 (60)	151 (34)	
Model 1^c^ OR (95% CI)	Reference	0.90 (0.61–1.33)	0.80 (0.53–1.21)	0.49** (0.31–0.78)	0.004
Model 2^d^ OR (95% CI)	Reference	0.90 (0.61–1.35)	0.79 (0.52–1.22)	0.48** (0.29–0.79)	0.005
Model 3^e^ OR (95% CI)	Reference	0.88 (0.59–1.33)	0.77 (0.50–1.20)	0.47** (0.28–0.79)	0.006

^a^Cholesterol levels are defined as quartiles in the complete case analysis

^b^The number of participants and events are stated as per the complete case analysis

^c^Model 1 is adjusted for age, sex, and education

^d^Model 2 is additionally adjusted for alcohol consumption, smoking, and body mass index

^e^Model 3 is additionally adjusted for hypertension, history of diabetes mellitus, use of cholesterol lowering medications, low density lipoprotein cholesterol, and triglycerides

**p < 0.01. Bold values denote statistically significant results

### Dementia

Compared to individuals included in complete case analysis, those with missing data for at least one of the variables included in the final models for dementia analyses were younger, more likely to be men, more likely to be smokers, have higher mean BMI, higher mean TG levels and lower mean HDL-C levels (eTable 1). The variables with the highest prevalence of missing data (36.9%–38.3%) were related to the health check-up.

The mean HDL-C level for participants in dementia analyses was 1.58 mmol/l (SD: 0.44 mmol/l). The mean age at the time of measuring cholesterol was 54.1 years (SD: 5.6 years) and the mean age at the time of the mental health screening was 73.2 years (SD: 5.7 years). At the mental health screening, 53 participants were diagnosed with dementia, of which 29 were complete cases.

Baseline characteristics for complete cases according to HDL-C quartile show that those in the highest quartile were less likely to be men, likely to consume more alcohol, less likely to be smokers, had lower mean BMI, and lower mean TG and LDL-C levels (Table 3).

**Table 3:** Baseline characteristics according to midlife serum high-density lipoprotein cholesterol (HDL-C) quartiles for dementia analyses

Characteristics	Serum HDL-C quartile (mmol/l)				*p* value^a^	Missing data (%)
	Quartile 1 (<1.29)	Quartile 2 (1.29–1.53)	Quartile 3 (1.55–1.81)	Quartile 4 (≥1.84)		
Proportion of all participants (%)	15.5	14.0	14.5	14.3		41.7
Age at screening [mean (years ± SD)]	73.6 ± 5.8	73.1 ± 6.2	72.9 ± 5.3	73.2 ± 5.5	n.s.	0
Men (%)	55.4	42.2	25.7	19.6	<0.001	0
Education (%)					n.s.	3.6
Junior high school	34.7	33.0	30.1	27.7		
High school	51.2	48.6	55.8	49.1		
College/vocational school, University or Other	14.1	16.7	14.2	23.2		
Alcohol consumption (%)					0.040	0
<150 g ethanol per week	78.5	73.4	88.5	80.4		
≥150 g ethanol per week	21.5	26.6	11.5	19.6		
Smoking status (%)					<0.001	4.1
Non-smoker	65.3	67.0	83.2	83.0		
Past smoker	9.9	14.7	7.1	10.7		
Current smoker	24.8	18.4	9.7	6.3		
Body mass index (kg/m^2^ ± SD)	24.5 ± 2.7	23.6 ± 2.6	23.4 ± 2.6	22.2 ± 2.4	<0.001	36.9
History of diabetes (%)	5.0	1.8	1.8	0	n.s.	0
Hypertension^b^ (%)	28.1	27.5	27.4	17.0	n.s.	36.9
Using cholesterol lowering medication (%)	0	0.9	2.7	2.7	n.s.	0
Triglycerides [mean (mmol/l ± SD)]	1.8 ± 0.9	1.3 ± 0.6	1.2 ± 0.6	1.0 ± 0.5	<0.001	37.4
LDL-C [mean (mmol/l ± SD)]	3.2 ± 0.7	3.2 ± 0.8	3.4 ± 0.9	3.0 ± 0.7	0.0019	38.3

*LDL-C* Low-density lipoprotein cholesterol

^a^Analysis of Variance (ANOVA) for age at screening, body mass index, and LDL-C; Welch’s ANOVA for triglycerides; Chi-square test for categorical variables

^b^Hypertension is defined as systolic ≥140 mm Hg or diastolic ≥90 mm Hg

Results of complete-case analyses are reported in eTable 3. Following imputation of missing values, the multivariable analysis showed no significant association with dementia for any of the HDL-C quartiles compared to the lowest the HDL-C quartile (Table 4). There was no significant trend for the association between HDL-C and dementia.

**Table 4:** The association between midlife serum high-density lipoprotein cholesterol (HDL-C) levels and dementia by multiple imputations using chained equations (n = 781, *m* = 100)

	HDL-C levels (mmol/l)^a^				
	<1.29	1.29–1.53	1.55–1.81	≥1.84	*P* for trend
Dementia					
No. (Events)^b^	121 (15)	109 (4)	113 (3)	112 (7)	
Model 1^c^ OR (95% CI)	Reference	0.34* (0.12–0.96)	0.33 (0.11–1.02)	0.45 (0.17–1.19)	n.s.
Model 2^d^ OR (95% CI)	Reference	0.32* (0.11–0.93)	0.33 (0.11–1.04)	0.41 (0.14–1.18)	n.s.
Model 3^e^ OR (95% CI)	Reference	0.34 (0.11–1.03)	0.38 (0.11–1.23)	0.40 (0.12–1.31)	n.s.

n.s. = non-significant

^a^Cholesterol levels are defined as quartiles in the complete case analysis

^b^The number of participants and events are stated as per the complete case analysis

^c^Model 1 is adjusted for age, sex, and education

^d^Model 2 is additionally adjusted for alcohol consumption, smoking, and body mass index

^e^Model 3 is additionally adjusted for hypertension, history of diabetes mellitus, use of cholesterol lowering medications, low density lipoprotein cholesterol, and triglycerides

*p < 0.05. Bold values denote statistically significant results

#### Post-hoc analyses

The small number of available cases in each of HDL-C quartiles 2–4 (Q2: *n* = 4; Q3: *n* = 3; and Q4: *n* = 7), and the comparable effect sizes (Q2: OR = 0.34; Q3: OR = 0.38; Q4: OR = 0.40) allowed for the collapse of the quartiles. Results of complete-case analyses on the binary HDL-C variable are reported in eTable 4. Multivariable adjusted analyses using multiple imputations showed a significantly reduced risk of dementia for those with high HDL-C (quartiles 2–4) compared to those with low HDL-C (quartile 1) (OR = 0.37, 95% CI, 0.16–0.88) (Table 5).

**Table 5:** The association between midlife serum high-density lipoprotein cholesterol (HDL-C) levels (Quartile 1 vs. Quartiles 2–4) and dementia by multiple imputations using chained equations (*n* = 781, *m* = 100)

	HDL-C level (mmol/l)^a^	
	<1.29	≥1.29
Dementia		
No. (Events)^b^	121 (15)	334 (14)
Model 1^c^ OR (95% CI)	Reference	0.38** (0.18–0.79)
Model 2^d^ OR (95% CI)	Reference	0.35* (0.16–0.78)
Model 3^e^ OR (95% CI)	Reference	0.37* (0.16–0.88)

^a^Cholesterol levels are defined as quartiles in the complete case analysis

^b^The number of participants and events are stated as per the complete case analysis

^c^Model 1 is adjusted for age, sex, and education

^d^Model 2 is additionally adjusted for alcohol consumption, smoking, and body mass index

^e^Model 3 is additionally adjusted for hypertension, history of diabetes mellitus, use of cholesterol lowering medications, low density lipoprotein cholesterol, and triglycerides

*p < 0.05; **p < 0.01. Bold values denote statistically significant results

## Discussion

This study shows that HDL-C concentrations at midlife are associated with cognitive function assessed 19 years later. Compared to individuals with very low levels of midlife HDL-C, 1) those in the highest HDL-C quartile at midlife have less than half the risk of MCI in late life, and 2) those with higher HDL-C levels (quartiles 2–4) at midlife have a significantly lower risk of dementia in late life. These findings are comparable to previous studies investigating the association between HDL-C and MCI,^5^ and dementia,^4,6^ respectively. There are, however, a number of studies which have failed to find any association between HDL-C and cognitive function,^7–10^ MCI,^11,12^ or dementia.^13–18^ One possible explanation for our significant associations is the timing of HDL-C measure in relation to the assessment of cognitive function. The 19 years between HDL-C measure and assessment of cognitive function in our study is a major difference compared to available research which has almost exclusively investigated the association between late-life HDL-C and late-life cognitive outcomes.^4–17^ Cholesterol levels decline prior to development of dementia,^19^ and may introduce bias to studies with concomitant late-life assessment of both HDL-C and cognitive function. It is important to consider early or midlife HDL-C measures given that the effect of lipids on cognition is most important before the age of 65,^33^ and that midlife lipid measurement can result in early lifestyle interventions in order to effectively delay the onset of dementia.^34^ In the present study only 13 individuals were above the age of 65 at the time of measuring HDL-C levels. Conversely, at the time of the mental health screening after 19 years of follow-up, only 13 individuals were younger than 65 years of age. The risk of bias due to age distribution in the present study can therefore be considered low.

The notable result of the present study is that midlife HDL-C is associated with both late-life MCI and late-life dementia. This strengthens the association between high midlife HDL-C levels and preserved cognitive function, but also makes a distinction of what constitutes low-risk midlife HDL-C levels for MCI and dementia, respectively. Whereas there was a significant linear trend with reduced risk of MCI with each increasing HDL-C quartile, the results for dementia indicate a marked reduction in the risk of dementia already from HDL-C quartile 2, with stable effect sizes throughout HDL-C quartiles 2–4. This suggests a possible threshold effect of midlife HDL-C on late-life dementia and emphasises that interventions and behavioural modifications which increase HDL-C, e.g., physical activity, moderate alcohol consumption, and smoking cessation^35^ are, for dementia purposes, of particular importance among those with the very lowest midlife HDL-C levels. Moreover, our finding of a linear trend between HDL-C and MCI contrasted with the threshold effect for dementia indicates that MCI shares some, but not all, of the HDL-C related risk associated with dementia. This finding is in accordance with the suggestion that MCI is a heterogeneous diagnosis,^36^ and may help explain why not all cases of MCI progress to dementia.^37^ Future studies are therefore encouraged to investigate the effect of midlife HDL-C on the progression from MCI to dementia.

One important consideration is whether low midlife HDL-C is a marker specific for late-life cognitive impairment, or whether it indicates an overall increase of lifestyle-related midlife cardiovascular risk factors with a subsequently increased risk of late-life cognitive impairment. Our analyses indeed suggest that low midlife HDL-C is a marker of cognitive decline independent from other known and important midlife cardiovascular risk factors such as smoking, overweight/obesity, hypertension, diabetes mellitus, LDL-C and TG. The biological mechanisms of such a protective effect of high HDL-C on both MCI and dementia, lie beyond the scope of the present study. In brief, the found associations could be due to: (1) genetic variants, e.g., I405V Cholesteryl ester transfer protein (CETP) which is associated with improved cognitive function, as well as higher HDL-C levels in the elderly,^3^ (2) vascular changes given that HDL-C is inversely associated with lacunar infarction independently of other CVD risk factors,^38^ and (3) interactions between HDL-C apolipoproteins and amyloid beta protein, a hallmark peptide of AD.^39^ Apolipoproteins promote the degradation of amyloid beta protein,^40^ as well as prevent its aggregation into amyloid.^41^

The use of cholesterol lowering medication could be considered a potential source of confounding in any study investigating the association between HDL-C and MCI and dementia. In the present study, only 3.1% and 1.5% of participants for MCI and dementia analyses, respectively, reported the use of cholesterol lowering medication at the time of HDL-C measurement. This could be considered a relatively small proportion, however, a recent meta-analysis of prospective studies found that the use of statins is associated with a reduced risk of all-cause dementia and MCI.^42^ Consequently, we adjusted for the use of cholesterol lowering medication in Model 3 of the complete case analyses, as well as following multiple imputations for both MCI and dementia outcomes.

There are a few limitations to this study. The long follow-up period has resulted in missing data due to death, as well as non-response to the original questionnaire and the mental health screening. Additionally, the proportion of missing values for HDL-C was approximately 40%. In 1995, HDL-C was not an obligatory measure during health check-ups in Japan, and the decision to measure HDL-C levels was decided by municipal policy or by health check-up physicians. This could possibly have resulted in a bias of the participants in the present study. However, instead of relying on complete case analyses, a potential source of biased estimates unless data is missing completely at random, we addressed the issue of missing data using multiple imputations. Overall, these results corresponded very well with those obtained from complete-case analyses. Second, HDL-C levels were measured at only one point in time. Although HDL-C may change over time owing to lifestyle decisions or illness, it would not impact the main aim of this study which was to focus on midlife HDL-C levels. Third, cognitive function was not assessed at baseline However, given the average time of 19 years between HDL-C measure and the mental health screening in 2014–2015, it is highly probable that the majority of participants would have had a normal cognitive function in 1995. Fourth, this study did not allow for the differentiation of the type of dementia. Finally, the study population resided in a rural area of Japan and may therefore not be representative of urban populations.

Despite these limitations, the study has a number of important strengths: This is a prospective study, in which the timing of HDL-C measure and the assessment of cognitive function were separated by approximately 19 years, thereby minimising any chance of reverse causation bias. Second, the detailed lifestyle questionnaire of the JPHC Study allows us to consider many important confounding factors for the found associations. Third, we have used multiple imputations to account for missing data and found robust associations between the exposure and the respective outcomes. Finally, MCI and dementia were classified by a trained psychiatrist in accordance with DSM-IV criteria.

## Conclusion

This study has found that high HDL-C levels in midlife are inversely associated with both late-life MCI and dementia. Midlife HDL-C thus has the potential to be used as a marker of late-life cognitive impairment.

## Supplementary information

Supplementary Information
