# Supplementary material for: The association between midlife serum high-density lipoprotein and mild cognitive impairment and dementia after 19 years of follow-up
Source: Transl Psychiatry. 2019 Jan 18;9:26. doi: 10.1038/s41398-018-0336-y (PMC6338778; doi:10.1038/s41398-018-0336-y)
Supplement: Supplementary file 1 — Supplementary Information [file 41398_2018_336_MOESM1_ESM.docx]

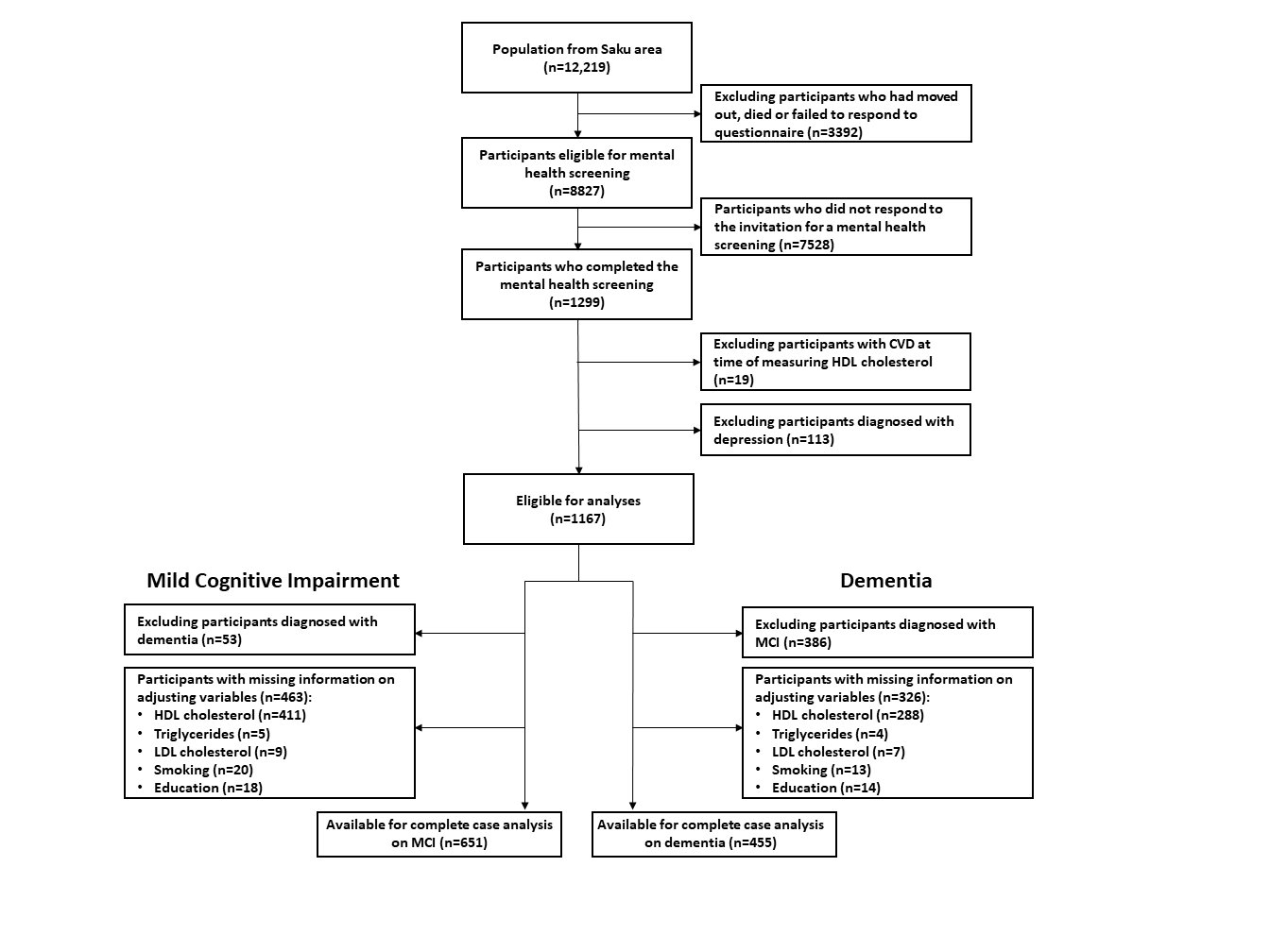


**eFigure 1. Flowchart of participant inclusion and exclusion for analyses on the association between midlife serum high-density lipoprotein cholesterol and mild cognitive impairment and dementia, respectively.**

| **eTable 1. Comparison of baseline characteristics between complete cases and participants with missing data for at least one variable** | | | | | | | | | |
| --- | --- | --- | --- | --- | --- | --- | --- | --- | --- |
|  | **MCI analyses** | |  |  |  | **Dementia analyses** | |  |  |
| **Characteristics** | **Complete cases** | **Missing data** |  | **p value**^1^ |  | **Complete cases** | **Missing data** |  | **p value**^1^ |
|  |  |  |  |  |  |  |  |  |  |
| **Proportion of all participants (%)** | 58.4 | 41.6 |  |  |  | 58.3 | 41.7 |  |  |
| **Age at screening [mean (years ± SD)]** | 73.5 ± 5.6 | 71.6 ± 5.2 |  | <0.001 |  | 73.2 ± 5.7 | 71.4 ± 5.2 |  | <0.001 |
| **Men (%)** | 38.3 | 49.5 |  | <0.001 |  | 36.0 | 45.1 |  | 0.011 |
| **Education (%)** |  |  |  | 0.02 |  |  |  |  | n.s. |
| Junior high school | 31.5 | 24.8 |  |  |  | 31.4 | 26.5 |  |  |
| High school | 51.6 | 53.5 |  |  |  | 51.2 | 53.7 |  |  |
| College/vocational school, University or Other | 16.9 | 21.7 |  |  |  | 17.4 | 19.8 |  |  |
| **Alcohol consumption (%)** |  |  |  | n.s. |  |  |  |  | n.s. |
| <150 g ethanol per week | 79.0 | 75.4 |  |  |  | 80.2 | 74.9 |  |  |
| ≥150 g ethanol per week | 21.0 | 24.6 |  |  |  | 19.8 | 25.2 |  |  |
| **Smoking status (%)** |  |  |  | <0.001 |  |  |  |  | 0.033 |
| Non-smoker | 75.1 | 66.0 |  |  |  | 74.5 | 68.0 |  |  |
| Past smoker | 10.1 | 8.4 |  |  |  | 10.6 | 9.5 |  |  |
| Current smoker | 14.8 | 25.5 |  |  |  | 15.0 | 22.5 |  |  |
| **Body Mass Index (kg/m^2^ ± SD)** | 23.5 ± 2.7 | 24.2 ± 2.6 |  | n.s. |  | 23.4 ± 2.7 | 24.4 ± 3.0 |  | 0.032 |
| **History of diabetes (%)** | 2.3 | 1.9 |  | n.s. |  | 2.2 | 1.8 |  | n.s. |
| **Hypertension**^2^ **(%)** | 26.3 | 25.0 |  | n.s. |  | 25.1 | 21.1 |  | n.s. |
| **Using cholesterol lowering medication (%)** | 3.1 | 1.7 |  | n.s. |  | 1.5 | 1.2 |  | n.s. |
| **HDL-C [mean (mmol/l ± SD)]** | 1.5 ± 0.4 | 1.4 ± 0.4 |  | 0.008 |  | 1.6 ± 0.4 | 1.4 ± 0.4 |  | 0.013 |
| **Triglycerides [mean (mmol/l ± SD)]** | 1.3 ± 0.7 | 2.6 ± 1.9 |  | <0.001 |  | 1.3 ± 0.7 | 2.5 ± 1.7 |  | <0.001 |
| **LDL-C [mean (mmol/l ± SD)]** | 3.2 ± 0.8 | 3.1 ± 0.8 |  | n.s. |  | 3.2 ± 0.8 | 3.0 ± 0.8 |  | n.s. |
|  |  |  |  |  |  |  |  |  |  |

___________________

^1^Analysis of Variance (ANOVA) for age at screening, body mass index, and LDL-C; Welch’s ANOVA for triglycerides; Chi-square test for categorical variables.

^2^Hypertension is defined as Systolic ≥140 mm Hg or diastolic ≥90 mm Hg.

HDL-C: High-density lipoprotein cholesterol; LDL-C: Low-density lipoprotein cholesterol; n.s.: non-significant

**eTable 2. Complete-case analysis for the association between high density lipoprotein cholesterol (HDL-C) levels
and mild cognitive impairment (MCI)**

|  |  | HDL-C Quartiles  (mmol/l) | | | |  |  |
| --- | --- | --- | --- | --- | --- | --- | --- |
|  |  | **Quartile 1 <1.29** | **Quartile 2 1.29-1.50** | **Quartile 3 1.53-1.76** | **Quartile 4 ≥1.78** |  | **P for trend** |
| **MCI** |  |  |  |  |  |  |  |
| No. (Events) |  | 175 (69) | 156 (62) | 169 (60) | 151 (34) |  |  |
| Model 1^1^  OR  (95% CI) |  | Reference | 1.01 (0.64-1.58) | 0.91 (0.58-1.44) | **0.47**  (0.28-0.78)** |  | 0.006 |
|  |  |  |  |  |  |  |  |
| Model 2^2^  OR  (95% CI) |  | Reference | 0.98 (0.61-1.55) | 0.87 (0.54-1.39) | **0.44**  (0.25-0.75)** |  | 0.004 |
|  |  |  |  |  |  |  |  |
| Model 3^3^  OR  (95% CI) |  | Reference | 0.93 (0.57-1.50) | 0.82 (0.50-1.34) | **0.39**  (0.22-0.70)** |  | 0.003 |

Bold values denote statistically significant results. **p<0.01;

^1^Model 1 is adjusted for age, sex, and education;
^2^Model 2 is additionally adjusted for alcohol consumption, smoking, and body mass index;

^3^Model 3 is additionally adjusted for hypertension, history of diabetes mellitus, use of cholesterol lowering medications, low-density lipoprotein cholesterol, and
triglycerides;

**eTable 3. Complete-case analysis for the association between high density lipoprotein cholesterol (HDL-C) levels
and dementia**

|  |  | HDL-C Quartiles  (mmol/l) | | | |  |  |
| --- | --- | --- | --- | --- | --- | --- | --- |
|  |  | **Quartile 1 <1.29** | **Quartile 2 1.29-1.53** | **Quartile 3 1.55-1.81** | **Quartile 4 ≥1.84** |  | **P for trend** |
| **Dementia** |  |  |  |  |  |  |  |
| No. (Events) |  | 121 (15) | 109 (4) | 113 (3) | 112 (7) |  |  |
| Model 1^1^  OR  (95% CI) |  | Reference | **0.23* (0.07-0.77)** | **0.20* (0.05-0.77)** | 0.49  (0.18-1.37) |  | n.s. |
|  |  |  |  |  |  |  |  |
| Model 2^2^  OR  (95% CI) |  | Reference | **0.22* (0.07-0.76)** | **0.20* (0.05-0.79)** | 0.47  (0.16-1.41) |  | n.s. |
|  |  |  |  |  |  |  |  |
| Model 3^3^  OR  (95% CI) |  | Reference | **0.23* (0.06-0.86)** | **0.23* (0.06-0.99)** | 0.44  (0.12-1.64) |  | n.s. |

Bold values denote statistically significant results. *p<0.05;

^1^Model 1 is adjusted for age, sex, and education;
^2^Model 2 is additionally adjusted for alcohol consumption, smoking, and body mass index;

^3^Model 3 is additionally adjusted for hypertension, history of diabetes mellitus, use of cholesterol lowering medications, low-density lipoprotein cholesterol,
and triglycerides;

n.s.: non-significant

**eTable 4. Complete-case analysis for the association between high
density lipoprotein cholesterol (HDL-C) levels (Quartile 1 vs Quartiles 2-4)
and dementia**

|  |  | HDL-C Quartile  (mmol/l) | | |
| --- | --- | --- | --- | --- |
|  |  | **Quartile 1 <1.29** | **Quartiles 2-4 ≥1.29** |  |
| **Dementia** |  |  |  |  |
| No. (Events) |  | 121 (15) | 334 (14) |  |
| Model 1^1^  OR  (95% CI) |  | Reference | **0.30** (0.13-0.70)** |  |
|  |  |  |  |  |
| Model 2^2^  OR  (95% CI) |  | Reference | **0.29** (0.12-0.69)** |  |
|  |  |  |  |  |
| Model 3^3^  OR  (95% CI) |  | Reference | **0.28* (0.10-0.77)** |  |

Bold values denote statistically significant results. *p<0.05; **p<0.01;

^1^Model 1 is adjusted for age, sex, and education;
^2^Model 2 is additionally adjusted for alcohol consumption, smoking, and body mass index;

^3^Model 3 is additionally adjusted for hypertension, history of diabetes mellitus, use of cholesterol
lowering medications, low-density lipoprotein cholesterol, and triglycerides;
